# Pharmaceutical brochures in Lebanon: do they meet WHO recommendations?

**DOI:** 10.1186/s12875-022-01930-5

**Published:** 2022-12-06

**Authors:** Yara Fadoul, Chadia Haddad, Jad Habib, Marouan Zoghbi

**Affiliations:** 1 Family Medicine Department, Saint Joseph University, Beirut, Lebanon; 2 Psychiatric Hospital of the Cross, Jal Eddib, Lebanon; 3 INSPECT-LB (Institut National de Santé Publique, d’Épidémiologie Clinique et de Toxicologie-Liban), Beirut, Lebanon; 4 School of Health Sciences, Modern University for Business and Science, Beirut, Lebanon

**Keywords:** Pharmaceutical, Brochures, Information, Criteria, Promotion

## Abstract

**Background:**

Drug promoting brochures can influence physicians prescription patterns. The validity of the info presented in these brochures is of major importance. World Health Organization (WHO) issued criteria to guarantee validity, equity and ethical presentation of data in medical brochures. This study aims to evaluate the quality and the validity of information presented in the pharmaceutical brochures distributed among family physicians in Lebanon.

**Methods:**

Pharmaceutical brochures were randomly collected at the family medicine center in Hôtel Dieu de France hospital in Beirut - Lebanon. These brochures were evaluated in reference to the WHO ethical criteria for medicinal drug promotion and on guidelines for quality assurance of the graphs, references, texts and illustrations.

**Results:**

Among the 60 brochures collected, only 4 fulfilled all the WHO ethical criteria, and 24 presented less than half the required criteria. Information concerning the drug safety are the least mentioned. Only 11.8% of the presented graphs are based on studies of high methodological level. Half of the brochures presented necessary information to identify studies references which are not always retrievable. Texts present mainly brand names instead of generic names and emphasize on information reflecting the drug efficacy.

**Conclusion:**

The pharmaceutical brochures in this study presented incomplete or invalid information. Prescribing physicians should be aware of the claims found in the brochures distributed by pharmaceutical companies and should be familiar with the principles of the evidence-based medicine to be able to critically appraise the validity of the reference studies and avoid the pitfalls in graphs reading.

**Supplementary Information:**

The online version contains supplementary material available at 10.1186/s12875-022-01930-5.

## Background

Pharmaceutical companies spend a lot of money on research and manufacturing of new drugs, but also on the marketing and promotion of these drugs [1]. Drug promotion effect on physicians attitudes and patterns of prescription has been the subject of an ongoing debate for over a decade now from professional, ethical and economic perspectives [2]. Drug promotion encourages doctors to prescribe particular medications and pushes pharmacists to administer pricey medications when less pricey ones would be superior in some situations which could lead to improper clinical use of some medications [3].

According to the World Health Organization (WHO), promotion refers to ‘all informational and persuasive activities by manufacturers and distributors, the effect of which is to induce the prescription, supply, purchase, and/or use of medicinal drug [4].

Pharmaceutical companies are highly involved in drug promotion, and the most widely used technique is the “Direct-to-physician (DTP)” [5]. This type of marketing is done by the mean of gifts, free samples, and drug brochures or through sponsored activities almost globally for continued medical education [6]. Most physicians put in a lot of effort, care deeply about their patients, and maintain their moral character. A massive inflow of funding into medicine from industry, particularly pharmaceutical corporations, has made it possible for them to be ready to put their own financial interests ahead of the health of patients [7].

Healthcare providers require adequate, comprehensive, easily accessible, and accurate medical information to help them make appropriate treatment or diagnostic decisions in line with rational prescribing practice [8]. The pharmaceutical industry represents through its various activities such as promotional activities (hiring of clinical expert to promote a drug) which is an important source of information about medications especially in developing countries [9, 10]. These promotions can be useful if subject to a critical evaluation and control assuring good quality and objective presentation of data [11] but may otherwise lead to biased and irrational prescriptions [12].

Studies have shown that pharmaceutical promotion may influence physicians’ behavior [13–16]. In a study evaluating factors associated with prescription quality in primary care, Figueiras et al. demonstrated that the prescription practice is influenced by the quality of the information source on the drug and that the relation between the physician and the pharmaceutical industry is an important modifier of the prescription [17]. Another proof of this potential influence has been shown in the Intercontinental Marketing Service (IMS) data, a study conducted in Abu Dhabi in 2010. That study showed that prescribing for more expensive products without established clinical data has been on the rise in recent years [18].

Several studies have been done that evaluate the reliability and validity of pharmaceutical brochures. A study done in Bangladesh found that 34% of the claims in a sample of 116 brochures for family physicians were misleading [19]. In Sri Lanka a considerable proportion of drug promotional materials collected in 2015 used poor quality scientific research as references [20]. A study done in In Iraq that was done among promotional drug brochures collected mainly from pharmaceutical exhibition have found that the information that is provided in medical brochures is biased [21]. A systematic review that identified 24 studies, reviewing advertisements from 26 countries, published between 1975 and 2006 have found that most of the advertisements provided the product’s brand and generic name, other information needed for rational prescribing, such as contraindications, interactions, side-effects, warnings and precautions were less commonly provided, and when supplied, were only available in the fine print [22].

The WHO has raised concerns about respect of ethics principles in drug and other pharmaceutical products promotion, and thus organized a conference around the following topic “WHO’s Ethical Criteria for Medicinal Drug Promotion” in 1988 [4]. The WHO stated that “Medical representatives should make available to prescribers and dispensers complete and unbiased information for each product discussed, such as an approved scientific data sheet or other source of information with similar content” [4].

Many countries adopted measures to regulate advertising by pharmaceutical companies [23]. The ministry of public health in Lebanon issued in July 2016 a decree (1/1356) about the good practice of pharmaceutical promotion. The decree stated that a national committee will be responsible of the application of the WHO ethical criteria for drug promotion. This decree also specified penalization procedures for the industries and the doctors in case of infringement.

The debate concerning the precision and reliability of the information given by medical representatives persists in both developed and developing countries [24, 25]. Studies have focused on investigating the influence of promotional information provided to physicians and prescribing behaviors, but the quality of the data and visual support presented in the promotional brochures has not been widely assessed [5].

We believe that information presented in medical brochures is an important source of information for physicians. Also, there is a need to inform patients using validated scientific data in order to facilitate patients’ involvement in making decisions about their own care. To our knowledge the quality of pharmaceutical brochures has not been evaluated in Lebanon. A well-designed brochure with good content can make a lasting impression on potential patients. Therefore, the objective of this study is to evaluate the quality and the validity of the information presented in pharmaceutical brochures distributed to promote drugs among family physicians in Lebanon.

## Methods

### Study design and setting

A cross-sectional study was conducted at the family medicine department in Hôtel-Dieu de France hospital in Lebanon between January 2020 and May 2020. Promotional pharmaceutical brochures were collected at one center, the family medicine department of Saint Joseph University at the Hotel Dieu de France hospital in Beirut. Brochures distributed in this center usually are comparable to all centers across Lebanon and they are issued by the marketing department, or the scientific bureau of the promoting companies based in Lebanon. Collection was done with the help of the physicians who received the representatives of the promoting companies in their offices for regular calls. Every time a brochure was delivered by the representative to the physician for promotion, and in a consecutive order, brochures were kept and addressed to the study coordinator. Some Brochures were catalogs about all available drugs at the company and contained no scientific data so we decided to retain brochures with scientific content about a single drug. Therefore, brochures promoting multiple drugs, cosmetics and medical devices were excluded.

### Procedure

A checklist was established to evaluate the pharmaceutical brochures (Supplementary file 1). The checklist includes five parts. The first part evaluates ethical criteria for medicinal drug promotion published by the WHO. Those criteria include the presence in the brochure of the following items: Name(s) of the active ingredient(s), Brand name, Content of active ingredient(s) per dosage form or regimen, Name of other ingredients known to cause problems, Approved therapeutic uses, Dosage form or regimen, Side effects and major adverse medicine reactions, Precautions, contraindications and warnings, Major interactions, Name and address of manufacturer or distributor, Reference to scientific literature as appropriate.

The four other parts evaluate the graphs, the cited references, the text and images appearing in the brochures respectively, inspired by a 2009 publication by WHO entitled “Understanding and Responding to Pharmaceutical Promotion”. The five parts was assessed equally according to the items of each part.

The graphs, when available, are evaluated according to the following criteria:

- Type of the information presented: Absolute Risk Reduction (ARR), Relative Risk Reduction (RRR), Number Needed to Treat (NNT) or other

#### Reference study is randomized and blinded

- Confidence intervals and power calculations are included when statistical significance is given
- Graphs are simple to read with appropriately labelled axes
- Graphs are obscured by other visual material
- Titles of graphs are clear and say explicitly what the graph is about
- The graph is reproduced exactly as it appeared in the original source
- Data in graphs are presented in a way that makes it easy to determine whether any differences are clinically meaningful

Cited references are evaluated on the following criteria:

- Citations contain all the information necessary to identify references
- Number of the cited references
- All cited references are retrievable
- Methodological type of the references: Meta-analysis, systematic review, randomized controlled trial, other
- Journal references come from peer-reviewed medical journals
- The research reported in the reference is financed by pharmaceutical company

The text appearing in the brochures is evaluated on the following criteria:

- Generic names are used as frequently as brand names
- The generic name is typed with the same size as that used for the brand name
- Claims reflect a Patient Oriented Evidence (POE) or a Disease Oriented Evidence (DOE)
- The information about safety is given the same prominence and placement as the information about effectiveness

The images are evaluated when present, on the following criteria:

- People portrayed in the advertisements reflect the racial and ethnic composition of people in our country
- Men and women are portrayed in advertisements as both patients and health-care providers in equal numbers
- The ways men and women are portrayed (as workers, facial expressions, body language, etc.) are similar.

The evaluation process was done by two independent reviewers. Every brochure was evaluated separately by both reviewers. Each reviewer was blinded to the evaluation made by the other. Results are then compared by the study coordinator after data entry. In case of discrepancy, the concerned brochure is re-evaluated to reach a consensus.

Brochures were classified as issued from national or multinational drug companies. National companies are companies established and working in Lebanon while multinational ones are those working in Lebanon through a scientific bureau with a headquarter in a European or American country.

### Statistical analysis

The SPSS software (version 21) was used to analyze data. For categorical variables, absolute frequencies and percentages were used, whereas for quantitative measurements, means and standard deviations (SD) were used.

We calculated the sample size based on the following assumptions. We estimated the total number of brochures published during the study period to about 200 and estimated the proportion of compliance to the WHO ethical standards to 50%. Sixty brochures are needed to reach a power of 90%.

For the descriptive analysis, a score was established for the ethical criteria based on the WHO ethical criteria cited before; Each criterion is assigned a score of ‘1’ if it is present and ‘0’ if it is absent. The final score is the sum of all the previous scores and ranges from 0 to 11.

The other items are analyzed separately as unique ordinal variables.

The data was considered normally distributed for continuous variables for samples of more than 30. Continuous variables were compared using a student test for mean comparison. Ordinal and nominal variables were compared using Chi square test for distributions.

A difference is considered statistically significant when the *p*-value is below 0.05.

## Results

A total of 60 brochures were evaluated.

Eleven (18.3%) of these brochures are issued by national pharmaceutical companies and 45 (75%) by multinational companies. Four brochures (6.7%) were classified as of unknown origin.

The analysis of WHO ethical criteria in the collected brochures is represented in Table 1. The total score established for these criteria varies between 2 and 11. Forty percent of the brochures scored 5 over 11 or less. The mean score is 6.8 over 11.

**Table 1:** Presence of WHO ethical criteria in the brochures (*N* = 60)

WHO ethical criteria	Total ***N*** = 60 (100%)	National companies ***N*** = 11 (18.3%)	Multinational companies ***N*** = 45 (75%)	***P***-Value
Name of active ingredient	59 (98.3%)	11 (100%)	44 (97.7%)	0.618
Brand name	60 (100%)	11 (100%)	45 (100%)	–
Content of active ingredient	47 (78.3%)	8 (72.7%)	36 (80%)	0.598
Other ingredients	22 (36.7%)	5 (45.4%)	15 (33.3%)	0.452
Therapeutic uses	47 (78.3%)	10 (90.9%)	33 (73.3%)	0.216
Dose	41 (68.3%)	8 (72.7%)	30 (66.6%)	0.700
Side effects	26 (43.3%)	5 (45.4%)	19 (42.2%)	0.846
Precautions, contraindications	24 (40%)	3 (27.2%)	19 (42.2%)	0.363
Major interactions	20 (33.3%)	2 (18.1%)	17 (37.7%)	0.219
Name and address of the manufacturer	32 (53.3%)	7 (63.6%)	23 (51.1%)	0.498
Reference to literature	30 (50%)	4 (36.3%)	23 (51.1%)	0.380

*WHO* World Health Organization

No statistically significant differences are identified comparing national and multinational drug companies concerning the presence of the WHO ethical criteria in the brochures.

### Graphs and visual illustrations

Seventeen (28.3%) brochures out of 60 presented data illustration by graphs. The graphs evaluation results are summarized in Table 2.

**Table 2:** Characteristics of the graphs found in 17 brochures

	Frequency (%)
Information presented ARR	2 (11.8%)
RRR	4 (23.5%)
ARR/ RRR	1 (5.9%)
NNT	0 (0%)
Other	10 (58.8%)
Randomized and blind study	2 (11.8%)
Confidence interval and power	2 (11.8%)
Simple to read and labelled axes	10 (58.8%)
Graph obscured	2 (11.8%)
Clear titles	9 (52.9%)
Exact reproduction	9 (52.9%)
Conclusion of significative difference easy to draw	3 (17.6%)

*ARR* Absolute Risk Reduction, *RRR* Relative Risk Reduction, *NNT* Number Needed to Treat

### References citation

Among the evaluated brochures, 34 (56.6%) of 60 cited the reference of the studies presented adequately. Most of the brochures mentioned 2 or 6 references (7 out of 60 or 11.6% respectively). Only five brochures (8.3%) are based on 10 references or more.

Among the evaluated brochures, 34 (56.6%) of 60 studies cited the reference of the studies presented adequately.43% did not cite any reference for the data published while 31% cited 1 to 5 reference and the remaining 26% cited more than 5 references .

The number of references cited in the brochures and the type of the reference studies is presented in Tables 3 and 4 respectively.

**Table 3:** Number of references cited in the brochures (*N* = 60)

	Frequency (%)
0	26 (43.3%)
1	5 (8.3%)
2	7 (11.6%)
3	1 (1.7%)
4	4 (6.7%)
5	1 (1.7%)
6	7 (11.6%)
7	2 (3.3%)
8	1 (1.7%)
9	1 (1.7%)
10	1 (1.7%)
11	2 (3.3%)
12	1 (1.7%)
16	1 (1.7%)

**Table 4:** Type of studies used in the brochures in which references are retrievable (*N* = 34)

Meta-analysis + RCT	1 (2.9%)
Meta-analysis + RCT+ other	1 (2.9%)
RCT	6 (17.7%)
RCT+ other	8 (23.6%)
Systematic review + RCT	1 (2.9%)
Systematic review + RCT+ other	1 (2.9%)
Other	16 (47.1%)

*RCT* Randomized Controlled Trials

### Text evaluation

The text evaluation was made for all the collected brochures. The generic name is used as frequently as the brand name in 28 (46.7%) brochures and is written in the same way (type and size) in only 3 (5%) brochures. The safety of the pharmaceutical product is mentioned on the same level as its efficacy in only 2 (3.3%) brochures. The type of the studies or results appearing in the brochures is presented in Table 5 and the results showed that Patient oriented evidence (POE) was presented in only 31.7% of the analyzed brochures.

**Table 5:** Type of the results reflected in the brochures (*N* = 60)

	Frequency (%)
POE	19 (31.7%)
DOE	19 (31.7%)
POE+ DOE	5 (8.3%)
None	17 (28.3%)

*POE* Patient Oriented Evidence, *DOE* Disease Oriented Evidence

### Images and photos

One or many images appear in 34 brochures out of 60 (56.7%). The ethnic and racial composition of our country is reflected in the images of 22 brochures out of 34 or 64.7%. Men and women are portrayed as both patients and health-care providers in 13 brochures out of 34 (38.2%) and are represented in the same way in 15 brochures out of 34 (44.1%).

## Discussion

This study shows that pharmaceutical brochures are mostly non-compliant to ethical and technical standards set by the WHO and may present biased information that lack solid support from scientific evidence. Only 6.6% of the brochures meet all the “WHO’s Ethical Criteria for Medicinal Drug Promotion” and 40% of them include less than half of these criteria. These results are consistent with those found in similar studies conducted in Nepal and Nigeria, showing that none of the brochures evaluated contain all the information required by the WHO [26–28]. A study done in Iraq, have found that the information that is provided in medical brochures is biased and mainly persuasive since it is mainly focusing on the positive aspect of drug therapy [21]. Also, a study done in United Arab Emirates (UAE) found that a misleading information was present in 5% of written pharmaceutical advertisement [29]. A study done in Texas among have found that 15% of the promotional marketing brochures presented data that was different from what was in the original published study [5]. In Germany in 2004, 94% of the brochures failed to be supported by scientific evidence [30]. This implies that drug promotional companies are more concerned with developing a business relationship with the treating physicians, which compromises the ethical educational aspect.

In Lebanon due to the presence of multiple crises, a shortage of pharmaceutical products occurred, with patients experiencing shortages of many drugs and several pharmacies began to report shortages of many products [31]. Lebanon imports their pharmaceutical products from outside the country such from U.S. companies, However, due to the economic crisis the importation have been reduced and the amount of available products have been shortened [32]. High production costs, a weak regulatory framework, and counterfeit drugs are additional challenges facing the pharmaceutical industry in Lebanon [32].

The least frequently found information are those concerning drug interactions (20%), the name of their active ingredients (22%), precautions or contraindications (24%) and side effects (26%). These results are consistent to those found in other studies [5, 8, 19, 28, 30, 33–36]. They are probably due to the fact that pharmaceutical companies tend to present information showing the drug efficacy and avoid highlighting the info on its safety of side effects. In fact, concerns have been raised earlier about studies sponsored by the pharma industry showing selective information about efficacy while hiding safety data by excluding it from the manuscript [37–39].

A graph is found in 28.3% of the brochures, but only 17.7% of them present the results as an absolute risk reduction. This way of data manipulation is a well known trick aiming to give the impression of a bigger effect size while the absolute risk reduction is the real measure of interest for a clinically informed decision [5]. .This can mislead physicians to conclude that a large difference in outcome occurs with the use of the promoted medication [40]. The graphs show otherwise low-quality studies knowing that only 11.8% of them only are randomized and blinded.

On another hand, the results of our study raise the issues of authenticity and reliability of the reference studies cited in the brochures. References are listed in almost half of the brochures but the corresponding studies are published and retrievable in 36.7% of them only .our results show that the majority of these references are of weak level of evidence. Previous studies by Van Winkelen et al. and Cooper and Schriger raised similar concerns about the validity reliability of the references. Ten percent of the brochures present data that are based on studies funded by pharmaceutical companies. A study by Cooper and Schriger in 2005 found that 58% of the original research cited in advertisements was sponsored by a pharmaceutical company or had a company affiliated author [41]. Sponsoring research by pharma is an important support for science development and sponsored studies are getting more common but the role and level of authority of the sponsor are not always well described and concerns raise from the sponsor ability to interfere with data analysis, the way results are presented and interpreted and the chances of publication especially in case of negative results.

Researchers warn that “Physicians should be cautious when drawing clinical decisions and conclusions based on data presented on brochures provided by pharmaceutical companies” [5]. This material aims mainly to promote a product instead of scientific appraisal and education [24, 42–44].

Images appear in 56.7% of the brochures. This rate is lower than that found in other Arab countries such as Iraq [21] or Libya [36]. Men and women are not portrayed in a similar way. This result supports the idea of gender bias in the images of promotional materials that was raised in other studies [45, 46]. The gender bias raises also the confusion about a possible selective efficacy of the promoted drug in one gender over the other.

No significant difference was found between brochures of national or multinational drug companies concerning the WHO ethical criteria. This result is discordant with other studies that found a significant difference favoring multinational industries [8]. Our study lacks of power to detect such a difference and bigger samples are needed to answer this question.

This study shows a clear gap in the medical brochures quality raising serious concerns about the potential negative influence on doctors prescriptions. The local authorities are requested to strictly apply regulations and to positively reinforce good practice. The order of physicians on another side can enhance physicians awareness to the possibility of bad information delivered through medical brochures and help physicians to acquire necessary skills to critically appraise evidence from the medical literature and red graphs with an informed eye to avid erroneous interpretation. We recommend adding literature reading skills and basic statistical presentation methods to the continuous medical education for physicians.

### Limitation

The brochures are evaluated by two reviewers only and the discrepancies in the evaluation are solved by a consensus and not by the opinion of a third reviewer. The sample size is small and lacks power for secondary analysis. The brochures are collected in one center of family medicine which could limit the generalizability of the results to other specialties, conditions (intra-hospital drugs for example) and geographic areas. Finally, our study is conducted in a university hospital; brochures of different or lower quality could be found in non-university sites.

## Conclusion

The promotional pharmaceutical brochures studied showed incomplete and invalid information. Prescribing physicians should be cautious toward the data presented in the brochures of pharmaceutical companies and should be aware of the common pitfalls in reading and using medical brochures as source of information for patient care.

## Supplementary Information

**Additional file 1.** Medical Brochures Checklist.

## Data Availability

The datasets generated and/or analyzed during the current study are not publicly available due to intellectual property/confidentiality issues but are available from the corresponding author on reasonable request.

## Abbreviations

WHO: World Health Organization
DTP: Direct-to-physician
ARR: Absolute Risk Reduction
RRR: Relative Risk Reduction
NNT: Number Needed to Treat
POE: Patient Oriented Evidence
DOE: Disease Oriented Evidence
SD: Standard deviation
RCT: Randomized controlled trial
