# Supplementary material for: Pharmaceutical brochures in Lebanon: do they meet WHO recommendations?
Source: BMC Prim Care. 2022 Dec 6;23:314. doi: 10.1186/s12875-022-01930-5 (PMC9724251; doi:10.1186/s12875-022-01930-5)
Supplement: Supplementary file 1 — Additional file 1. Medical Brochures Checklist. [file 12875_2022_1930_MOESM1_ESM.docx]

**Supplementary file 1**

**Medical Brochures Checklist:**

- Drug Name:
- Pharmaceutical Company Name:
- Pharmaceutical Company Type (national, multinational):

1. OMS Ethical Criteria: (Place a Checkmark if available or an X if not available)

- Name(s)of the active ingredient(s) using either international non proprietary name (INN) or the approved generic name of the medicine
- Brand name
- Content of active ingredient(s) per dosage form or regimen
- Name of other ingredients known to cause problems
- Approved therapeutic uses
- Dosage form or regimen
- Side effects and major adverse medicine reactions
- Precautions, contraindications and warnings
- Major interactions
- Name and address of manufacturer or distributor
- Reference to scientific literature as appropriate

B. Graphs and Data evaluation:

Does the brochure contain a graph or a data? Yes No

If the previous answer is Yes, complete the following checklist: (put a circle over the right answer)

- Is information presented as:
- Absolute Risk Reduction (ARR)
- Number Needed to Treat (NNT)
- Relative Risk Reduction (RRR)
- Does the advert indicate if a study was randomized and blinded? Yes No
- When statistical significance is given are confidence intervals and power calculations included?

Yes No

- Are graphs simple to read and do they have appropriately labelled axes? Yes No
- Are graphs obscured by other visual material? Yes No
- Are the titles of graphs clear and do they explicitly say what the graph is about? Yes No
- If the graph comes from an article or another source is it reproduced exactly as it appeared in the original source? Yes No
- Are data in graphs presented in such a way as to make it easy to determine whether or not any differences are clinically meaningful? Yes No

C. References evaluation:

- Do citations contain all of the information necessary to identify references?

Yes No

- Number of cited references: …
- Are all references cited retrievable? Yes No
- Methodological type of references: (indicate the number of references for each type)
- Meta-analysis
- Systematic review
- Randomized controlled trial
- Other
- Do journal references come from peer-reviewed medical or pharmacy journals? Yes No
- Did the company finance the research reported in the reference? Yes No

D. Text evaluation:

- Are generic names used as frequently as brand names? Yes No
- Is the type of the generic name the same size as that used for the brand name?

Yes No

- Do the claims reflect a: Patient oriented endpoint

Disease oriented endpoint

- Is information about safety given the same prominence and placement as information about effectiveness? Yes No

E. Pictures and Images evaluation:

Does the brochure contain a picture? Yes No

If the previous answer is Yes, complete the following checklist: (put a circle over the right answer)

- Do the people portrayed in the advertisements reflect the racial and ethnic composition of people in our country? Yes No
- Are both men and women portrayed in advertisements as both patients and health- care providers in equal numbers? Yes No

Are the ways that men and women are portrayed (as workers, facial expressions, body language, etc.) similar? Yes No
